# Decision-making process during collective movement initiation in golden snub-nosed monkeys (*Rhinopithecus roxellana*)

**DOI:** 10.1038/s41598-019-57191-3

**Published:** 2020-01-16

**Authors:** Chengliang Wang, Ruliang Pan, Xiaowei Wang, Xiaoguang Qi, Haitao Zhao, Songtao Guo, Yi Ren, Weiwei Fu, Zirui Zhu, Baoguo Li

**Affiliations:** 1 Shaanxi Key Laboratory for Animal Conservation, College of Life Sciences, Northwest University, Xi’an, 710069 China; 2 Shaanxi Institute of Zoology, Xi’an, 710032 China; 3 Center for Excellence in Animal Evolution and Genetics, Chinese Academy of Sciences, Kunming, 650223 China

**Keywords:** Animal behaviour, Behavioural ecology

## Abstract

Collective decision-making is important for coordination and synchronization of the activities among group-living animals and the mechanisms guiding such procedure involve a great variety of characteristics of behavior and motivation. This study provides some evidence investigating collective movement initiation in a multi-level social band of the golden snub-nosed monkeys (*Rhinopithecus roxellana*) located in the Mts. Qinling, China. We collect 1223 datum records relevant to decision initiation from six OMUs. The results indicate that collective movement initiation could be divided into two continual but relatively independent processes: decisions on moving direction and movement implementation. In both processes, adult individuals are more likely to initiate the decision-making, while other adults vote on initiator’s preference, with a threshold, a supporting number required for a success. Thus, voting behavior and quorum fulfillment contribute to a successful decision-making. Adult individuals play important role in making decisions for moving direction and implementation. For a successful collective movement initiation, the individuals being more central in grooming network initiate decisions more frequently than the others, and attract voters more easily. Furthermore, following the initiation, at least four positive voters are required for a direction decision and at least three positive voters are needed for the decision on movement implementation, which could be considered as the threshold of quorum numbers required for a successful decision. This study has provided some very interesting information and scientific evidence in understanding social structure and behaviors of the nonhuman primates with a social structure very similar to humans’. Thus, some results can directly be referred to the comprehension of human social structure and behavior.

## Introduction

Many animal species have evolutionarily developed their unique social structures under which there are a number of social groups that differ enormously in size, composition and permanence. Group-living animals usually have more benefits in survival and developments than those living alone, through reducing predatory risk, sharing resource, strengthening defense force, cooperative foraging, shared vigilance, and information transfer^1^. However, individuals within the groups vary in their nutritional requirements, habitat occupation, and ability to monopolize resources, and thus they have different motivations in decision making. If some individuals always prefer their own needs or motivate above others within a group, discord may cause group fragmentation and the loss of group-living advantages^2^. Thus, individuals must maintain a group cohesion and synchronize activities efficiently in order to continue social benefits of group-living. Under such a situation, some individuals have to try to keep a balance between their benefits and maintaining cohesion within a group. For example, when a wild bonobos (*Pan paniscus*) group travels from one location to another, some individuals might have to shorten their resting time when the group starts to travel, while others might have to wait until the group finishes feeding before they can depart. Thus, compromises are particularly common in collective group movement^3–6^.

Collective movement decision-making has gained much attention over the past several decades and demonstrates a wide diffusion across species with alternative evolution levels and social structures, ranging from eusocial insects^7,8^ to birds^9^, mammals^10,11^ and primates^12–14^. There are, however, generally two kinds of decision patterns in the process of collective movement decision-making. Animals with large groups, such as a flock of birds or school of fish, individuals make collective decisions follow a simple rule to synchronize movement and maintain cohesiveness, adjusting their direction and speed referring to neighboring individuals, known as a self-organizing system^3,15^. However, regarding stable and cohesive social groups, such as those of lions, wolves, and primates, group members are able to communicate directly, with one or several individual(s) leading group’s decision-making and others following the decision, which is called leadership^16^.

Researchers have classified such a leadership into two forms: (1). personal leadership that occurs when a single individual uses its high dominant social status or unique experience to lead the group with an “unshared decision”^3,17^. This occurs to mountain gorillas (*Gorilla beringei beringei*), where the silverback male directs the group by heading to his preferred direction and other group members have no choice but follow the leader^18^; (2). “shared leadership” in which any group member makes contribution to collective movement and the related decision making, and there is an intermediate form between the two extremes, called “partial leadership”, some group members or sub-groups lead the procedure of decision-making. Partial leadership is the most popularly observed among mammals^19^, such as in hamadryas baboons (*Papio hamadryas*), wherein the initiating and decision-making individuals are males, mainly older ones, with the leaders of one-male units (OMUs) that have the most influence in the decision-making process^20^.

Several factors have been considered to make contribution to different types of leadership. Individual attributes can affect leadership^15^, such as dominant rank (e.g., rhesus macaque *Macaca mulatta*^21^; wolf *Canis lupus*^22^; feral horse *Equus ferus caballus*^23^), sex preference^24–26^, age advantage (e.g., African elephants, *Loxodonta Africana*^27^), reproductive status (e.g., lactating females^28–30^**)**, and experience (e.g., homing pigeons *Columba livia*,^4,9,31^**)**. Social attributes also can influence the leadership^15,32^, for instance social organization, individuals with strong dominant hierarchies are most likely to have personal leadership^9,21,22,26,33^, and those with weak social status normally result to the shared leadership^13,21,34^; social interaction, individuals with strong social affiliations tend to be followed more often^4,35,36^; and individual importance within the social network, individuals with higher eigenvector centrality usually initiate successful departures than social individuals^32^.

A successful collective movement requires several steps for a final achievement, and a pre-departure period is playing an important role to influence group movement^37^. During the pre-departure period groups make a decision on moving direction or departure time, followed by group members^6,26^. Two types of pre-departure behavior patterns which are vocalization and voting behavior contribute to decision-making. Regarding the decision on departure time, group members can display their motivations about when to move by simply increasing activity or exhibiting a specific behavior (e.g., vocalizations in Canadian goose, *Branta Canadensis*^38^; mountain gorilla, *Gorilla beringei beringei*^26,39^**)**. As for decision on moving direction, however, the choice is more complex than simple departure time and involves voting for different direction preferences. For example, when all group members wish to move, and different directions could be preferred, group members must vote for which is their preferred direction^20,26,40^. These pre-departure behaviors are typical of a shared consensus process that exists in almost all sociable animal species. They usually allow each individual to express its intention to decision making and ultimately plays a role in the launching of departure^6,12,41^.

A collective decision-making process involves not only the voting behavior but also a quorum threshold^7,8,29,42^. For example, regarding hamadryas baboon (*Papio hamadryas*) males repeatedly attempt to exhibit their preferred direction, and influence other group mates, and finally the entire group move to the direction selected once that male has gained the most support^20,43^. African buffalo (*Syncerus caffer*) also exhibit similar voting behavior^40^, Adult cows initially display certain direction orientating behaviors until the group eventually departs for a new grazing location, with a departure in the direction of the most frequently observed orientation. In both cases, a quorum offers a simple and efficient way for animals to achieve a fast and accurate collective decision^8,42,44–46^.

In collective group movement, a follower is often as important as a initiator in regard to a successful decisions. However, to work out an accurate threshold number for a successful decision is quite difficult, since the initiation may be viewed as failure if no individuals follow^21,25,47^. A mean number of the followers that determines a successful group movement can vary considerably among species, which may also be changed within a given season or due to the variation of resource abundance^35,48^. Early studies (wild *Propithecus* and *Eulemur*^49^) set a threshold of 50% of group members following for a successful movement^49^. However, other studies on other primates indicate that a certain number of followers (quorum) is required for a successful decisions on movement (e.g., five in chacma baboons *Papio hamadryas*^2^; three in Verreaux’s sifaka *Propithecus verreauxi*^25^). This quorum is an important parameter in decisions making^50^, and is considered a tradeoff between speed and accuracy during the decision-making process^3^. In general, a threshold of approximately three followers seems to be sufficient to elicit a group movement^48^. Since this number is big enough to provide sufficient protection against predators or generate collective knowledge for orientating within the home range and detecting resources.

The golden snub-nosed monkey (*Rhinopithecus roxellana*) is one of the colobine species endemic to China. It inhabits at high-altitude (1800–3000m) mountainous regions with remarkable seasonal variation of the forests features in central China. It feeds principally on leaves, fruits, buds, bark, lichen, and seeds^51^. This species is distinct from the other Asian colobines by forming large multi-level societies composed of more than 100 individuals^52^. The primary social and reproductive unit in its society is the OMU (One Male Unit). Members of an OMU travel and live together, high cohesive association, and remain spatially separated from the other OMUs. An OMU consists of one adult male, several breeding females, and their offspring^53,54^. Dominant hierarchies exist within the group, which are different between OMUs. However, there is no clear or consistent rank order among adult individuals within the same OMU^52^, and both intersexual bonds and female-female kin-bonds contribute to the maintenance and cohesion^55^.

Thus, through this study we analyze the mechanisms of how collective movement in the golden snub-nosed monkeys is conducted by focusing on how such movement is initiated within wild OMUs; who initiates the decision and participates in decision making; how the leadership is formed; and what kind of factors play important role in decision-making. Accordingly, we proposed the following predictions:

1. Because there is no consistent dominant hierarchies among the individuals within the OMU, we suppose that a shared leadership exists so that any group member is supposed to have a role in leading collective movement.
2. Due to the affiliative relationships within the group, individuals with higher eigenvector centrality values in grooming networks would be more likely to initiate movement than other individuals with lower values.
3. Since the number of the followers is important for a successful decision-making, we assume that quorum size is at least three followers elicit group movement.

## Methods

All research reported in this manuscript adhered to the Integrative Zoology Principles for the Ethical Treatment of Non Human Primates. All research protocols reported in this manuscript were reviewed and approved by the Chinese Academy of Science. Our research received clearance from, and complied with the protocols approved by animal care committees of Wild Life Conservation Society of ShaanXi Province, China. All research reported in this manuscript adhered to the legal requirements of the Guanyinshan National Reserve, China, in which the work took place.

### Study site and subjects

This study was conducted in Dapingyu region of the Guanyinshan National Nature Reserve (GNNR), which is located on southern slopes of the Mts. Qinling, Shaanxi Province, China (107°52′−108°02′E,33°20′−33°44′N) with an elevation of 1150–2574 m above sea level. Vegetation structure varies following the variation of the altitude and is dominated by deciduous broadleaf forest under 1500 m; coniferous and deciduous broadleaf mixed forest between 1500–2200 m; and coniferous forest above 2300 m. The area has a semi-humid montane climate. Average annual rainfall is approximately 924 mm, and average annual temperature is 11.5 °C, with a minimum of −14.3 °C in January and a maximum of 36.4 °C in July. The monkeys are the only residents in the region.

We targeted on Dapingyu Troop (DPT) in GNNR that has been observed since 2009. It composed of breeding band, all-male band, and several solitary males. The home range of the breeding band encompasses 15 km^2^ in mountainous forest, and is extremely difficult to be followed while they were crossing steep ravines and mountainous terrains. From 2010, we got habituated with this band using semi-provision^53^, in which individuals were provisioned with approximately 200 g of corn and apples per monkey per day for over a period of 20 days/month.

This targeted breeding band was consisted of 66 individuals belonging to six OMUs, with six adult males, 26 adult females (16 of them gave the birth), five sub-adult individuals, and 13 juveniles. (Table 1**)**

**Table 1:** The composition of study band.

Age-sex categories	CH unit	CM unit	DG unit	GG unit	PT unit	TY unit	Proportion of category
Adult male	1	1	1	1	1	1	12.00%
Adult female with infant	3	2	3	3	2	3	32.00%
Adult female without infant	2	2	1	0	1	4	20.00%
Sub-adult	1	0	2	0	2	0	10.00%
Juvenile	1	0	3	2	2	5	26.00%

Note: CH, CM, DG, GG, PT, and TY units are the six targeted OMUs.

### Behavioral definitions

#### Initiator

A siting group member turns its body to a certain direction, lasting for at least 10 s during the pre-departure period, or an individual moving at least more than 5m beyond the periphery of the group to a certain direction, without the same movement from the other group members.

#### Voter

Individuals turn their body to face the direction driven by the initiator during the pre-departure period, or individuals moving to a certain distance in the direction driven by the initiator before the whole OMU is moving.

#### Pre-departure period

When the whole targeted OMU members are exhibiting free activity, one individual turns its body toward a certain direction, indicating getting ready for departure. While the first individual moves a certain distance, the pre-departure period ends.

#### Quorum

Minimum number of the voters showing the same behavior as the initiator, and launching collective group behavior.

#### Direction decision-making (DDM)

OMU members driving the direction to move during the pre-departure period.

#### Movement implementation decision-making (MDM)

OMU members deciding how to move when the pre-departure period finishes.

#### Successful direction decision

When the direction initiated by initiator has been followed by the whole group members.

#### Decision on successful movement implementation

After 50% of the OMU members have joined the movement process.

### Behavioral data collection

We conducted the research from April to December 2017, in which we started. The observations from 09:00 am to the time when the whole band left the provision to their sleeping site each day^55^. In turn, we observed one of the six OMUs per day.

When the targeted OMU in a given day finished feeding and returned to the trees surrounding the provision area, we recorded behavior via scan sampling^56^, with the information on the activity of all the members recorded. Scans were repeated at 5-min intervals for as long as the OMU was in view. If the whole OMU had moved out of view, the observation site was adjusted to allow a continued data collection next day.

During the afternoon (15:00 to 18:00 pm), while the band was preparing to leave the provision area, we recorded OMU’s movement via a digital camera (Canon G10, Canon Corporation) using all occurrences sampling^56^, so that were able to get the data based on the recorded behaviors, including times of the events, sequence and name of each individual involved with collective movement.

### Data analysis

We classified the subjects into five age-sex categories: adult males (AM), adult females with infants (AFI), adult females without infants (AFN), sub-adults (SA), and juveniles (Juv). As infants were always carried by their mothers during collective movement, any data related to the infants were excluded.

### Social status of each individual within the OMUs

As grooming is an important interactive social behavior, it was used to evaluate social relationships among the individuals within each OMU. We used the following formula to calculate grooming index for each individual daily:

$$Index=\frac{[{F}_{A}(B)]+[{F}_{B}(A)]}{[F(A)+F(B)]}$$

where, F(A) and F(B) are the total number of scans for grooming A and B, respectively; F_A_(B) is the number of the scans in which B is the grooming partner of A when A was scanned; and F_B_(A) is the number of the scans in which A is the grooming partner of B when B was scanned.

To evaluate the social status regarding a specific individual within the OMU, we calculated it’s the eigenvector centrality coefficient according to grooming index.

### Relative leadership distribution

We compared absolute frequency of initiations among the categories using homogeneity chi-square test (the number of initiations per category is divided by a total number of initiations of all the categories). However, absolute frequency does not reflect the probability of a category initiated based on the number of individuals per category, thus, we compared relative leadership among the categories using homogeneity chi-square test (the number of initiations observed per category is divided by the ratio of individuals of this category within a given OMU. This corrected number is then divided by the sum of all corrected number of initiations for all categories). Relative leadership is ranged from 0 to 1, where 0 indicates that the category never leads the group and 1 indicates that the category always leads the group^6,12^.

### Influencing factors on relative leadership

We then clarified whether individual attributes had an effect on the frequency of successful or failed initiations. To do so, we used the generalized linear model (GLM) to examine the effects of age, sex, lactation state, affiliative relationships, and eigenvector centrality on initiation.

### Link between direction and movement initiation

Association rule analysis is a popular and well recognized approach in discovering the interesting relationships among the variables collected. Such interesting relationship is usually expressed as X => Y, where X and Y are two disjoint subsets of all available in the database. X is regarded as the antecedent or LHS (left hand side) and Y as the consequent or RHS (right hand side). Interesting rules have to satisfy the constraints on the measures of significance and interestingness, the best-known constraints are minimum thresholds of support and confidence degrees, which are calculated with the following equation.

Support degree=$\frac{number(XY)}{number(all\,sample)}$, where XY represents the number of combined XY, all the samples in the database. Confidence degree is defined as when X appears, the probability that Y appears. Confidence degree (X => Y)=$\frac{P(Y|X)}{P(X)}$.

We analysed the relationship between direction decisions and the decision on movement implementation with association rule analysis. We tried to established association rule between the two relative independent decisions with the Apriori Algorithm model. We set the initiator in direction decision as LHS and the initiator movement implementation decision as RHS. The minimum thresholds on support and confidence are set as 0.1.

### Quorum of successful collective movement

We confirmed the minimum number of voters required for a successful direction and the decision on movement implementation, and then performed survival analysis, in which “resultant event” in the model was set as the individual who joined the decision-making.

All statistical tools were from RStudio v1.1.44 (RStudio Team, 2018), with two-tailed test, and with *p* < 0.05 was the threshold of significance. Average values were expressed as means ± SD. The packages which used in our analysis as follow: the social network analysis used igraph package, survival analysis used survival and survminer packages, the Association rules analysis used Arules package and the GLM analysis used based package in R software.

## Results

We recorded 1,223 direction decision initiations (average 203 ± 16.61 for six OMUs), and 865 successful direction decisions that triggered movement implementation initiation (average 144.17 ± 13.85 for six OMUs). Regarding decision-making process, 4.48 ± 1.18 individuals were required for a successful direction decision, and 3.6 ± 1.30 individuals were required for a successful movement implementation decision. As for successful direction decisions, 344 (39.76%) were initiated by adult males, 340 (39.31%) by adult females with infants, 178 (20.58%) by adult females without infants, and 3 (0.35%) by sub-adults. With regard to successful movement implementation, 328 (42.21%) were initiated by adult males, 283 (36.42%) by adult females with infants, 149 (19.18%) by adult females without infants, and 17 (2.19%) by sub-adults. Juvenile category was never observed initiating either decision.

### Social relationships within targeted OMUs

**Figure 1:**
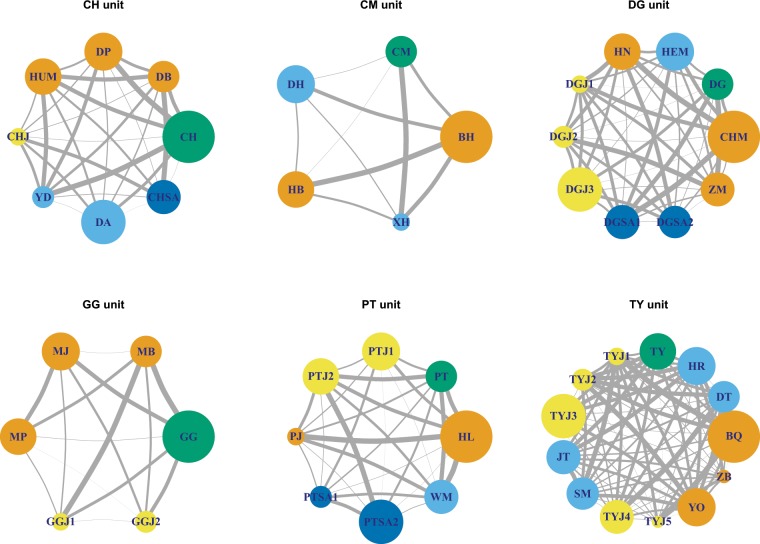
Social relationships among the individuals within six targeted OMUs. Circle represents individuals. Different categories are colored differently. Size of the circles indicates the coefficient degree of eigenvector centrality. Gray lines represent the relationship between individuals, and its thickness reflects the variation of grooming index. Size of the nodes represents the variation of eigenvector centrality coefficient.

The eigenvector centrality coefficient for each individual was calculated from the grooming index. Eigenvector centrality coefficients showed a decreasing rank: adult males> adult females > sub-adults > juveniles (Fig. 1).

### Relative leadership driving the initiations of direction and movement implementation

As for decision making on direction and movement implementation, the frequency of successful initiations is not equally distributed across different age-sex categories (Direction decision: relative frequency: *χ*^2^ = 670.43, df = 4, *p* < 0.001; relative leadership: *χ*^2^ = 333.96, df = 4, *p* < 0.001; Movement implementation decision: relative frequency: *χ*^2^ = 575.4, df = 4, *p* < 0.001; relative leadership: *χ*^2^ = 439.57, df = 4, *p* < 0.001). Adult males are the most successful initiators for both decisions, illustrated by the highest likelihood across all categories (28.13% in direction initiation, and 37.91% in movement implementation initiation) and the highest likelihood of leadership success (42.75% in direction initiation, and 54.01% in movement implementation initiation). Conversely, sub-adults are the least successful initiators for both decisions (relative leadership is only 0.4% in direction initiation and 0.34% in movement implementation initiation).The juvenile category never initiated successful decisions (Table 2).

**Table 2:** Numbers of successful and failed initiations, and the decision on relative leadership in direction and movement implementation.

Initiator (category)	Direction decision			Movement implementation decision		
	Success	Failure	Leadership	Success	Failure	Leadership
Adult male	0.281	0.054	0.427	0.379	0.046	0.540
Adult female with infant	0.278	0.093	0.158	0.327	0.031	0.175
Adult female without infant	0.146	0.049	0.133	0.172	0.023	0.147
Sub-adult	0.002	0.026	0.004	0.020	0.001	0.034
Juvenile	0.000	0.070	0.000	0.000	0.000	0.000

The frequencies of the failed initiations for both decisions are also not equally distributed across the different age-sex categories (Direction initiation: relative frequency: *χ*^2^=53.223, df=4, *p*<0.001; Movement implementation initiation: relative frequency: *χ*^2^=67.114, df=4, *p*<0.001). Furthermore, relative leadership is not significantly different among the categories (Direction initiation: leadership: *χ*^2^=3.546, df=4, *p*=0.471; Movement implementation initiation: leadership: *χ*^2^=7.001, df=4, *p*=0.136).

### Influencing factors on direction and movement success

The GLM was used to test the factors affecting successful direction and movement implementation initiation. As for the decisions on direction and movement implementation, initiator identity (individual attribute) has no significant effect on a successful consensus decisions, and the number of the voters is the key factor driving decision-making processes (Table 3). Regarding the decisions on direction and movement implementation, initiator and voters jointly decide whether there should be collective movement. On the other hand, eigenvector centrality coefficients in grooming network are positively correlated with a successful direction initiation (Table 3).

**Table 3:** Effects of age, sex, and eigenvector centrality on initiation.

	Direction initiation				Movement implementation initiation			
	Estimate	Std.	z	*P*	Estimate	Std.	z	*P*
Intercept	−9.210	1.264	−7.285	<0.01	−12.893	4.278	−3.014	<0.01
AFN	0.050	0.345	0.145		−1.049	1.021	−1.027	
AM	−0.933	0.506	−1.843		1.601	1.668	0.959	
Juv	−17.704	871.595	−0.020		—	—	—	
SA	−0.066	0.952	−0.070		0.953	2.292	0.416	
Direction	2.114	0.152	13.940	<0.01	6.247	1.295	4.824	<0.01
ECC	9.932	2.701	3.677	<0.01	3.584	7.955	0.451	

Note: AFN: adult females without infant; AM: adult males; Juv: juveniles; SA: sub-adulst; ECC: eigenvector centrality coefficient

### Number of voters required for a successful decision-making

**Figure 2:**
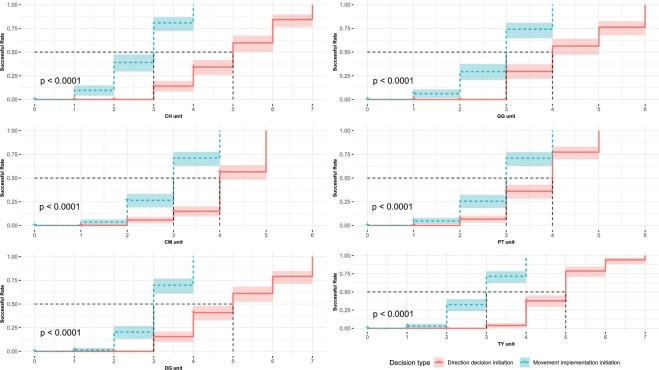
The relationship between decision-making success rate and number of the voters. Red line means direction decision, green line indicates movement implementation decision and dotted line represents median of success rate.

Survival analysis was used to determine the number of the voters required for a successful decision on direction and movement implementation for six targeted OMUs (Fig. 2). As for a direction decision, initiation usually fails if voters’s number is under four, and there is a more than 50% of success rate if voters’ number is increased from four to five. With regard to the decision on movement implementation, less voters are required for a success. For example, such a rate is ~35% if voters’ number is less than three; ~65% with at least three voters; and 100% with more than four voters **(**Fig. 2**)**.

### Link rule between direction selection and movement implementation

**Figure 3:**
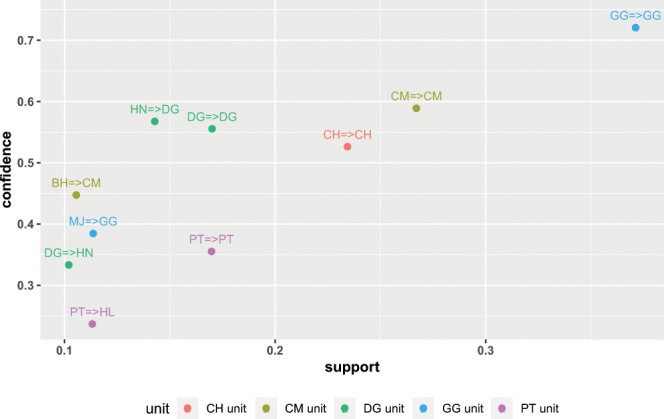
Rules linking direction and decision on movement implementation. X=>X presents the initiator leading to the decisions on moving direction and movement implementation. Colors of points represent different targeted units.

According to the data on successful collective movement within each targeted OMUs, 10 rules have been found among OMUs; they are GG unit 2, CH unit 1, CM unit 2, DG unit 3, PT unit 2 and without rules from TY unit. Within those rules, 50% (5 rules) from adult males =>adult males, 30% (3 rules) from adult females with infants =>adult males, and 20% (2 rules) from adult males => adult females with infants. (Table 4, Fig. 3).

**Table 4:** Ten link rules between direction and movement implementation decision making.

Target unit	LHS	link	RHS	Support	Confidence
CH unit	CH	=>	CH	0.23	0.53
CM unit	CM	=>	CM	0.27	0.59
CM unit	BH	=>	CM	0.11	0.45
DG unit	DG	=>	DG	0.17	0.56
DG unit	HN	=>	DG	0.14	0.57
DG unit	DG	=>	HN	0.10	0.33
GG unit	GG	=>	GG	0.37	0.72
GG unit	MJ	=>	GG	0.11	0.38
PT unit	PT	=>	PT	0.17	0.36
PT unit	PT	=>	HL	0.11	0.24

**Note**: LHS means the initiator of direction decision making; RHS means initiator of movement implementation decision making.

## Discussion

The results based on nonhuman primates and illustrated in the tables and figures have provided very interesting values and information for us to address the issues proposed in this study. We found that collective movement initiation could be divided into two continual but relatively independent processes; collective movement direction decision occurs before OMU departure, which triggers a decision on which direction to move; the decision on collective movement implementation occurs once the direction has been decided, which triggers the decision on how to implement movement to the direction chosen.

In both processes, leadership is not equally distributed; adult individuals (especially adult males) are more likely to initiate collective direction and movement implementation decisions than any other age-sex categories within the OMU (Table 2). Nevertheless, a successful initiation depends not only on the initiating adult individual, but also on other adult voters. Thus, regarding the monkeys studied, leadership is not formed by a single adult individual that is able to elicit the followers^48^, but consisted of different adult individuals exhibiting alternative behaviors to be involved with a consensus decision (Fig. 2). Leadership is shared among adult categories; each having a potential chance to participate in consensus decisions, either as an initiator or voter. In contrast, although sub-adults and juveniles can try to initiate a decision, their attempts are almost never successful due to non-support from adult individuals, except for a sub-adult female individual who successfully initiated three direction decisions (1.89%) and 17 movement implementation decisions (11.49%) within the PT OMU. At the beginning of the study, she was categorized as a sub-adult. She was older than the other sub-adult individuals while she was involved with the decision and gave birth in the following breeding season. In other words, she was actually an adult female. If a successful initiation of collective movement can be regarded as a success of the leadership, the golden snub-nosed monkeys expresses a system of partial leadership^18^. Thus, our results make us exclude Prediction 1.

Partial leadership is most commonly observed among mammals^15,19^. Regarding some species, the leadership is distributed among individuals, particularly the old ones (e.g. African elephants, *Loxodonta Africana*^27^)with better knowledge about the environment^27,57^. As for other species, leadership is distributed among dominant individuals (e.g., rhesus macaque *Macaca mulatta*^21^; wolf *Canis lupus*^22^) or specific sexs (white-faced capuchin monkeys^24^). Previous studies have reported that social relationships can directly affect the distribution of leadership(Tibetan macaques^32^**)**. In our study, individual attributes from sex and age categories influence successful decision-making differently (Table 3). Eigenvector centrality coefficient that quantifies the attraction of a given individual to the others in the group^32,58^ is positively correlated with leadership distribution (Table 3), implying that among monkey individuals whose who have better social association with others are more likely to initiate a successful decisions, and attract followers during collective movement. Thus, results supports the Prediction 2.

Although a collective movement initiation is composed of two relatively independent decision-making processes, our study shows that the decisions on collective movement are primarily governed by voting behavior and quorum fulfillment (Fig. 2). As for a direction decision in pre-departure period, an initiator (an OMU adult) turns its body in a particular direction indicating its preferred choice for the movement coming; another adult member in OMU would then vote for this direction by exhibiting the same behavior; and if the number of voting individuals reaches the quorum, the direction decision is made (Fig. 2). With regard to the decision on movement implementation, an initiator (OMU adult) walks a certain distance and waits for the responses from other adults; and if the number of the followers reach a quorum, the entire OMU moves (Fig. 2). Voting behavior and quorums have been reported from many animal species. For example, African buffalo and hamadryas baboon^29^. In wolves, gorillas, and Tonkean macaques, collective decisions are made by social amplification or selective mimetism, a group decision reaches when the voter number exceeds the minimum quorum^41^. However, what we found on the golden monkeys are somewhat different; an initiator’s behavior is more similar to that for recruitment and a final decision depends on the number of the voters, but not a majority of the OMU.

We also found that regarding a collective movement initiation, two decision processes are involved in a continuous procedure. This is somehow different from what found in the Tonkean macaques: the transition mechanism linking pre-departure with departure decisions is a continuous procedure in decision-making for a collective movement^12^. This mechanism is primarily governed by mimetism to notify behaviors during the pre-departure period, with a quorum combined with selective mimetism required at departure. These two processes decide the time and direction to which individuals will move^29^. In our research, we conducted association rule analysis to determine the link rule between the direction and the decision on a movement implementation. The related results (Table 4 and Fig. 3) indicate that the two decision processes are linked by an adult initiator. That is, the adult individual who initiates a successful direction decision is more motivated to initiate the decision on movement implementation.

Although a quorum is considered to be a simple and efficient way to achieve collective decisions^41^, a threshold number for a positive decision outcome may vary substantially among species, and may be changed seasonally or resource abundance^35,48^. Earlier research classified movement initiation as a failure no individuals followed the initiator^21,25,47^. Based on the threshold of 50% group members are required for successful movement^49^, however, other studies have indicated that the number of followers (quorum) required for a successful movement decision can vary within a range and is likely taxon-specific^35,48^ (e.g., five in chacma baboons, *Papio hamadryas*^2^; three in Verreaux’s sifaka *Propithecus verreauxi*^25^). The demand of a quorum is likely to provide sufficient protection against predators or collective knowledge to orientate within the home range and detect resources^48^. Furthermore, a quorum can promote decision-making and make it more accurate^46^. With regard to the golden monkeys, we found that a quorum number affects decision accuracy and success^3^. This is perhaps due to the fact that the golden snub-nosed monkeys are facing less pressure from the predation in the habitats with condense forests^52^ and their food resources are seasonally abundant or uniformly distributed^52,53^. In other words, aggregation for defense and foraging chances seem not to be the main impact factor on a quorum selection. On the other hand, the times for making a decision on moving direction and movement implementation are not significantly different among the six target OMUs. This implies that decision speed is not an important factor required for a quorum number. In regard to decision accuracy, however, a quorum is required for the golden monkeys, although voters’number not a fixed one (ranging from three to five). As long as individuals have employed a quorum rule, the threshold could vary greatly, with less effect on decision making, and the whole group could reach a solution accurately. As a result, our study allow us to accept Prediction 3.

## Supplementary information

Dataset 1.
